# Cold-chain transmission, asymptomatic infection, mass screening, vaccine, and modelling: what we know so far for coronavirus disease 2019 control and experience in China

**DOI:** 10.1515/mr-2021-0036

**Published:** 2022-02-25

**Authors:** Wenjing Gao, Liming Li

**Affiliations:** Department of Epidemiology & Biostatistics, School of Public Health, Peking University, Beijing, China; Peking University Centre for Public Health and Epidemic Preparedness & Response, Beijing, China

Coronavirus disease 2019 (COVID-19) pandemic has continued to spread rapidly across the world. In the past nearly two years, there have been over 267 million confirmed COVID-19 cases including over five million deaths globally reported to WHO [1]. Facing this unprecedented public health crisis which we have not seen in a century, global experts rapidly expand scientific knowledge on this new virus, to track the spread and virulence of the virus. Different countries develop response strategies and practices tailored to their own specific epidemiological situations, resources, and values of individuals living in their countries [2].

Although first reported, quick containment of COVID-19 was achieved in China. It set an inspiring example for the world. China experienced two stages for COVID-19 response [3]. The first stage began from the first case reported in Wuhan at the end of 2019, and continued until Wuhan ended lockdown on Apr 8, 2020. From then on, the main challenges in the second stage changed from the interruption of widespread community transmission to the prevention of sporadic outbreak resurgence from cases overseas. China’s practice of breaking community transmission, followed by prevention of severe acute respiratory syndrome coronavirus 2 (SARS-CoV-2) reintroduction from imported cases overseas show that established public health measures will remain the best tool [4]. That is, COVID-19 can be controlled and even eliminated with non-pharmaceutical interventions (NPI) alone (e.g., early detection, early reporting, early isolation, masks, social distancing, and handwashing). Prof Hao and his colleagues from Sun Yat-Sen University will introduce a meta-analysis on the reproductive number of COVID-19, which reflects the COVID-19 transmission dynamic. According to the scale of reproductive number reduction, they conclude that comprehensive interventions and lockdowns were most effective in the pandemic control.

In the second stage of COVID-19 response, several sporadic COVID-19 re-emergence outbreaks in China were linked to cold-chain foods or its packaging. Apart from air-borne transmission, indirect contact transmission might become a route of infection, by which SARS-CoV-2 was easily transmitted from overseas. Prof Li from Huazhong University of Science and Technology will review the cold-chain related COVID-19 epidemics in China, analyze their potential mechanisms and introduce China’s experience in interruption of cold-chain transmission.

Asymptomatic and pre-symptomatic infection is a main feature in which SARS-CoV-2 is different from SARS-CoV and Middle East respiratory syndrome coronavirus (MERS-CoV). Compared to these two viruses, the proportion of asymptomatic and pre-symptomatic infections in all cases infected seems higher for COVID-19. Considering their infectiousness and “hidden” distribution in the population, the roles of infections without symptoms should be estimated in this pandemic [5]. We will review the population size, infectiousness, as well as China’s strategy and measures in asymptomatic and pre-symptomatic cases finding and management. With the surge of new SARS-CoV-2 variants, the character of asymptomatic and pre-symptomatic infections may have changed [6]. We also provide related evidence on the changing trend.

Mass screening is an important strategy for asymptomatic and pre-symptomatic case finding. However, it is controversial due to its huge social and economic costs, although it has been increasingly used to test close contacts, high risk employees, even all population to ascertain individuals infected. Prof Wang and his colleagues from Beijing Center for Disease Prevention and Control (CDC) will introduce the history of mass screening, define its scope and application scenarios, and discuss the challenges in using this approach. They conclude that with a comprehensive plan supported by strong testing facilities and capabilities, rapid SARS-CoV-2 testing might help life return to normal more quickly. This article will be published in the coming issue.

The development of COVID-19 vaccine has been much quicker than that of any other vaccines. Over 300 vaccine candidates were in clinical or pre-clinical development [7]. Despite of effectiveness that vaccines showed in preventing infections and fatal illness, those challenges including long-term immunity, variants and vaccine inequity still exist. Prof Song and his colleagues from Guangdong CDC will review COVID-19 vaccine studies, focusing on vaccine types, efficacy, efficiency, protection against new variants, breakthrough infection, safety, and vaccination progress. This article will also be published in the coming issue.

Transmission dynamics models are critical tools in COVID-19 epidemiological parameter estimation, prediction, and intervention evaluation. But we must recognize that it is essential to differentiate between what models can and cannot forecast [8]. Prof Chen and his colleagues from Nanjing Medical University will introduce the history of disease transmission, summarize transmission dynamics models, highlight their key contribution in pandemic control. The limitations and challenges of transmission dynamics models will be listed in their review.

Global researchers have accumulated plenty of knowledge in COVID-19 response, which cannot be covered one by one in this collection. We focus on summarizing new characteristics of SARS-CoV-2 and China’s practice, through which we hope to provide up-to-date evidence support for the public health professionals. We believe that the experience in China will have significant public health implications for the world at present, especially for those countries where COVID-19 epidemic is in low-level. Meanwhile, we must acknowledge that for this new virus there has been progress but still far from enough. To conquer COVID-19, we need to continue to work together to make greater achievements on the basis of evidence researchers have accumulated.
